# Efficacy and Safety of Paromomycin in Treatment of Post-Kala-Azar Dermal Leishmaniasis

**DOI:** 10.1155/2014/548010

**Published:** 2014-03-06

**Authors:** Shyam Sundar, Anup Singh, Anurag Tiwari, Saurabh Shukla, Jaya Chakravarty, Madhukar Rai

**Affiliations:** Department of Medicine, Institute of Medical Sciences, Banaras Hindu University, Varanasi 221005, India

## Abstract

*Background*. Post-kala-azar dermal leishmaniasis (PKDL) plays an important role in maintaining endemicity of visceral leishmaniasis and its transmission. Treatment regimens for PKDL are toxic and require 3-4 months of hospitalization. These long and arduous regimens result in extensive noncompliance. There is an urgent need to develop a safe, effective, and acceptable regimen for the treatment of PKDL. Paromomycin (PM) has been recently approved in India for treatment of visceral leishmaniasis (VL); hence we tested its efficacy in patients with PKDL. *Methods*. In this exploratory study, 31 patients with PKDL aged 10 years and above were administered PM 11 mg/kg daily intramuscularly for 45  days and followed up for one year. *Results*. Out of 31 patients, 7 patients were lost to followup at 1  year and 9 (37.5%) got cured with complete disappearance of lesion, while 15 (62.5%) showed no improvement by per protocol analysis. *Conclusion*. Cure rate with 45 intramuscular injections of PM was unacceptably low though there was no serious side effect of the drug. Whether paromomycin can be used in multidrug therapy to shorten the duration of treatment should be the next logical step for investigation.

## 1. Introduction

Post-kala-azar dermal leishmaniasis (PKDL) is a dermal manifestation of* Leishmania donovani* infection and often follows resolution of clinical visceral leishmaniasis (VL). However, it may also manifest without prior history of VL in a small minority of patients [1]. PKDL is characterized by macular, papular, or nodular lesions or a mixture of them. It is quite common in Sudan (occurring in >50% of patients with VL), where it may occur concurrently or follows immediately after an episode of VL and heals spontaneously in majority of patients [2], whereas in the Indian subcontinent it occurs in 2–20% of patients, 6 months to several years after an episode of VL [3]. In a recent trial the prevalence of confirmed PKDL cases was 4.4 per 10 000 individuals and 7.8 if probable cases were also considered [4].

Several treatment regimens have been recommended for the treatment of PKDL in India, for example, 120 days of parenteral sodium stibogluconate (20 mg/kg body weight) [1] or three courses of 20 daily infusions of amphotericin B with an interval of 20 days in between the courses [5]. These inordinately long parenteral regimens invariably lead to either nonacceptance or poor compliance. In the last decade two new antileishmanial compounds, miltefosine and paromomycin, have been approved for the treatment of VL in India. There is a report about the efficacy of miltefosine at a higher dose 50 mg TID for 60 days (with a need to extend to 90 days if required) in small number of PKDL patients in Bangladesh [6]. However, paromomycin is yet to be tested for the treatment of PKDL. Of all the antileishmanial drugs, paromomycin is considerably cheaper and is produced in India [7]. In this study, we tested its efficacy and safety in patients with PKDL.

## 2. Material and Methodology

The study was carried out at the Kala-Azar Medical Research Center, Muzaffarpur, the field site of the Institute of Medical Sciences, Banaras Hindu University. Patients with PKDL were enrolled between October 2007 and August 2009 and followed for one year after treatment. The study was approved by the Institutional Ethics Committee, and written informed consent was taken from all enrolled patients and by the parents of patients of <18 years of age. Patients of both sexes, aged more than 10 years, with skin lesions of PKDL (nodules, papules, plaques, or macules) identified by a qualified and trained doctor at Kala-Azar Medical Research Center, Muzaffarpur, with or without history of an episode of VL in the past, were included in the study. Slit-skin scraping of lesions from each patient was taken. Taking into account the decreased slit-skin smear parasite and culture positivity in diagnosis of PKDL we did only PCR for making the diagnosis [8]. The immune-chromatographic rK39 strip test was performed in serum of all the patients. Only PCR and k39 positive confirmed cases were included in the study. However, for followup and treatment efficacy only clinical parameters were used.

Patients with platelet count <100000/mm^3^, total leukocyte count (TLC) <2500/mm^3^ and hemoglobin (Hb) <8.0 g/100 mL, hepatic enzymes >3 times the upper limit of normal range, serum bilirubin >2 times the upper limit of normal range, and serum creatinine or blood urea nitrogen (BUN) above normal range were excluded from the study. Patients with any noncompensated or uncontrolled condition, such as active tuberculosis, malignant disease, severe malaria, HIV, or other major infectious diseases, lactation, pregnancy, or inadequate contraception in females of childbearing potential for the treatment period, were also excluded from the study.

Once eligible for enrollment, patients were given daily paromomycin 11 mg/kg (base) intramuscularly for 45 days. Clinical parameters like vital signs were assessed daily, and hematological and biochemical assessments were done on days 15, 30, and 45 of treatment. For monitoring the adverse events, except nephrotoxicity, common toxicity criteria of National Cancer Institute were used [9]. If there was toxicity of grade 3 and above, the treatment was discontinued and the subject was removed from the study and offered rescue treatment. Nephrotoxicity was defined as an increase in serum creatinine that was either double the baseline levels or more than 2.0 mg per deciliter [177 *μ*mol per liter].

### 2.1. Assessment of Efficacy

Efficacy was assessed by decrease in size or disappearance of the lesion. Cure was defined as complete disappearance of skin lesion(s) after treatment, as reported by the patient and assessed by the trained physician at 12-month followup.

### 2.2. Rescue Treatment

Those patients who failed treatment in the form of no response and increase in number and size of lesion at 1-year followup and those patients in whom treatment was stopped due to adverse events were offered rescue treatment with three 20-day courses of amphotericin B in doses of 1 mg/kg given 20 days apart [5].

### 2.3. Statistical Analysis

The data were analyzed by using SPSS-16 version. The data were checked for assumption of normality. Comparison of means was done by using paired Student's* t*-test for paired data. A *P* value less than 0.05 (<0.05) was considered as statistically significant.

## 3. Results

Thirty-one patients with PKDL were included in the study out of which three patients did not give a history of a prior episode of VL. The baseline characteristics of patients are shown in Table 1. The median duration of interval between treatment of VL and development of PKDL was two years. Amongst the previously treated patients for VL, 22 patients were treated with sodium stibogluconate (SSG), 3 patients with amphotericin B and miltefosine each, and 1 patient with paromomycin.

Among the various forms of skin lesions 27 patients had exclusively hypopigmented macular patches. Among the rest four patients one had nodular lesion on face/chin with hypopigmented macular lesion over trunk, arms, and lower limb, and the other had hypopigmented macular and nodular lesions over the whole body. One patient presented with erythematous lesion over face while one patient presented with nodular and erythematous lesion on face with hypopigmented macular lesion over the whole body.

### 3.1. Safety

PM was well tolerated by all patients without any significant derangement in hematological and biochemical parameter. None of the patients complained of tinnitus and hearing loss. 14 patients (45.2%) complained of pain at injection site which was the most common side effect noted.

Posttreatment clinical, biochemical, and hematological parameters were similar to the baseline values except slight improvement in the platelet count (Table 1).

### 3.2. Efficacy

**Figure 1:**
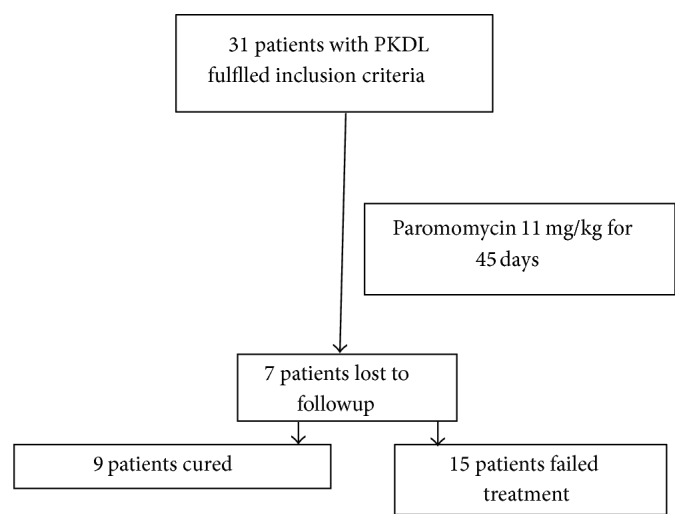


At one-year followup, out of 31 patients, 9 (37.5%) were cured with complete disappearance of lesion while 15 (62.5%) patients showed no improvement; rather there was increase in lesion and appearance of nodular lesions in 11 patients. 7 patients were lost to followup. All the patients who improved were having macular lesions. Among three patients who had nodular lesions, two were lost to followup and one failed the treatment (Figure 1).

## 4. Discussion

PM is very effective in the treatment of VL with a cure rate of ~95% [7]. These results prompted us to use PM for the treatment of PKDL. Since dose/duration of every antileishmanial drug in PKDL is 2-3 times that used for VL, we arbitrarily selected a regimen of PM for 45 days (~2 times of VL) for the treatment of PKDL. Though 45 days of PM treatment did not result in any noticeable adverse event except the expected injection site pain, the cure rate with this relatively better tolerated regimen of PM was unfortunately far from satisfactory. Since these patients were admitted in the hospital for the entire duration of treatment, and PM injection was administered by nursing staff; compliance was ensured. In India cure rates are 64–92% with SSG in doses of 20 mg/kg per day for 120 days [2]. SSG, being a toxic drug with high volume (10–12 mL) of intramuscular injections given in long regimen of 120 days, makes acceptance of this regimen very poor. Similarly 60 injections of amphotericin B given over 100 days pose a real threat of nephrotoxicity [5]. Unfortunately, nearly every trial for PKDL consists of small number of patients with no randomization.

Clinical response may differ according to the type of lesions in PKDL; nodules and papules disappear in 120 days, macules in 200 days [10]. Thus it becomes imperative to follow these patients for a sufficiently long duration to see the long term effects of treatment. Though the cure rate was unacceptably low, it had noticeable activity and cured one-third of the patients. Future studies using paromomycin with another antileishmanial drug like miltefosine could yield successful short duration regimen for the treatment of PKDL and should be tested. It is also important to know that the tolerance to the drug was excellent even though the drug was used for twice the duration of that used in VL.

The unresponsiveness of PM in PKDL which is very effective for treatment of VL highlights that there is a lot to be still discovered about the mechanism and pattern of response of PM against different manifestations of leishmaniasis.

## Figures and Tables

**Table 1:** Baseline characteristics and comparison of biochemical parameters at days 0, 30, and 45∗.

Parameters	Followup (days)			*P* value	
	Day 0 (*n* = 31)	Day 30 (*n* = 31)	Day 45 (*n* = 31)	0 versus 30 days	0 versus 45 days
Age (years) ± SD	17.10 ± 8.05				
Sex, number (%)					
Male	22 (70.9)				
Female	9 (29.1)				
Weight (kg) ± SD	41.48 ± 13.67				
Hb (gm%) ± SD	12.33 ± 1.94	11.89 ± 1.89	12.03 ± 1.85	0.536	0.502
Wbc (/mm^3^) ± SD	9056.64 ± 3190.26	9748 ± 3604.82	9525.92 ± 3558.80	0.328	0.456
Platelets (/mm^3^) ± SD	216258.06 ± 81649.24	246760 ± 84001.13	248111.11 ± 84413.51	0.438	0.029∗∗
Serum creatinine (mg/dL) ± SD	0.67 ± 0.16	0.6496 ± 0.18	0.7 ± 0.22	0.62	0.54
SGPT (IU) ± SD	29.38 ± 13.50	30.19 ± 8.53	31.80 ± 10.57	0.796	0.456

^*^Calculated by using paired Student's *t*-test.

^**^Significant at 5% level of significance.

SD: standard deviation.

## References

[1] Thakur C. P., Kumar K., Sinha P. K. (1987). Treatment of post-kalar-azar dermal leishmaniasis with sodium stibogluconate. *British Medical Journal*.

[2] Zijlstra E. E., Musa A. M., Khalil E. A. G., El Hassan I. M., El-Hassan A. M. (2003). Post-kala-azar dermal leishmaniasis. *Lancet Infectious Diseases*.

[3] Thakur C. P., Kumar K. (1992). Post kala-azar dermal leishmaniasis: a neglected aspect of kala-azar control programmes. *Annals of Tropical Medicine and Parasitology*.

[4] Singh R. P., Picado A., Alam S. (2012). Post-kala-azar dermal leishmaniasis in visceral leishmaniasis-endemic communities in Bihar, India. *Tropical Medicine & International Health*.

[5] Thakur C. P., Narain S., Kumar N., Hassan S. M., Jha D. K., Kumar A. (1997). Amphotericin B is superior to sodium antimony gluconate in the treatment of Indian post-kala-azar dermal leishmaniasis. *Annals of Tropical Medicine and Parasitology*.

[6] Ramesh V., Katara G. K., Verma S., Salotra P. (2011). Miltefosine as an effective choice in the treatment of post-kala-azar dermal leishmaniasis. *British Journal of Dermatology*.

[7] Sundar S., Jha T. K., Thakur C. P., Sinha P. K., Bhattacharya S. K. (2007). Injectable paromomycin for visceral leishmaniasis in India. *The New England Journal of Medicine*.

[8] Mondal D., Nasrin K. N., Huda M. M. (2010). Enhanced case detection and improved diagnosis of PKDL in a Kala-azar-Endemic area of Bangladesh. *PLoS Neglected Tropical Diseases*.

[9] Cancer Therapy Evaluation Program (1999). *Common Toxicity Criteria of the DCTD, NCI, NIH, DHHS*.

[10] Thakur C. P., Kumar K. (1990). Efficacy of prolonged therapy with stibogluconate in post kala-azar dermal leishmaniasis. *Indian Journal of Medical Research A*.

